# Probing for congenital nasolacrimal duct obstruction: a systematic review and meta-analysis of randomized clinical trials

**DOI:** 10.5935/0004-2749.20210005

**Published:** 2025-02-02

**Authors:** Joyce Godoy Farat, Silvana Artioli Schellini, Regina El Dib, Felipe Gasparini dos Santos, Roberta Lilian Fernandes Sousa Meneghim, Eliane Chaves Jorge

**Affiliations:** 1 Department of Ophthalmology, Otorhinolaryngology and Head and Neck Surgery, Faculdade de Medicina de Botucatu, Universidade Estadual Paulista “Júlio de Mesquita Filho”, Botucatu, SP, Brazil; 2 Oculoplastic Division, King Khaled Eye Specialist Hospital, Riyadh, Saudi Arabia; 3 Institute of Science and Technology, Universidade Estadual Paulista, São José dos Campos, SP, Brazil; 4 Department of Community Health and Epidemiology, Dalhousie University, Halifax, Canada

**Keywords:** Lacrimal duct obstruction/congenital, Lacrimal duct obstruction/therapy, Infant, Obstrução dos ductos lacrimais/congênito, Obstrução dos ductos lacrimais/terapia, Lactente

## Abstract

**Purpose:**

Lacrimal probing is the treatment of choice for congenital nasolacrimal duct obstruction that does not have a spontaneous resolution; however, there is no consensus about the best time for probing and if it is superior to other therapies. The present study aimed to evaluate the effectiveness of lacrimal probing compared with other treatments/no intervention to treat congenital nasolacrimal duct obstruction.

**Methods:**

A systematic review of literature in PubMed, EMBASE, CENTRAL, clinicaltrials. gov, and LILACS databases up to December 2019 was performed. Randomized clinical trials that enrolled children diagnosed with congenital nasolacrimal duct obstruction and undergoing lacrimal probing were considered. Data extraction and a risk of bias assessment were conducted independently and in duplicate. The overall quality of evidence for each outcome was conducted using the Grading of Recommendations, Assessment, Development, and Evaluation classification system.

**Results:**

Four randomized clinical trials involving 423 participants were eligible. No statistically significant differences were observed in resolution rates between early probing and observation/late probing (two studies; risk ratio 1.00 [95% confidence interval 0.76-1.33]; p=0.99; low certainty evidence). One study reported better resolution rates with bicanalicular silicone stent intubation compared with late probing in the complex congenital nasolacrimal duct obstruction cases subgroup (risk ratio 0.56 [95% confidence interval 0.34-0.92]; p=0.02; moderate certainty evidence).

**Conclusions:**

Low certainty evidence suggests that early probing has the same success rate as late probing. Evidence of moderate certainty suggests that late probing has a lower success rate than bicanalicular silastic intubation in patients with complex congenital nasolacrimal duct obstructione.

## Abstract (pt)

**Objetivo:**

A sondagem lacrimal tem sido o tratamento de escolha para a obstrução lacrimonasal congênita que não apresenta resolução espontânea. Contudo, não há consenso sobre qual é a melhor época para a realização da sondagem e se ela é melhor do que outras terapias. O objetivo foi avaliar a efetividade da sondagem lacrimal no tratamento da obstrução lacrimonasal congênita**. Método**: Uma revisão sistemática da literatura foi realizada usando as plataformas eletrônicas PubMed, EMBASE, CENTRAL, clinicaltrials.gov e LILACS até o período de dezembro de 2019. Foram considerados ensaios clínicos randomizados envolvendo crianças com obstrução lacrimonasal congênita submetidas a sondagem lacrimal. A extração dos dados e avaliação do risco de viés foram feitas por dois autores independentemente. A análise da qualidade da evidência para cada desfecho foi realizada por meio do sistema GRADE (*Grading of Recommendations Assessment, Development and Evaluation*).

**Resultados:**

Quatro ensaios clínicos randomizados foram incluídos, envolvendo 423 participantes. A metanálise mostrou que não houve diferença estatística na resolução da obstrução lacrimonasal congênita entre o grupo submetido à sondagem lacrimal precoce e o submetido à observação/sondagem tardia (2 estudos; risco médio 1.00 [intervalo de confiança de 95% 0.76, 1.33] p=0,99, I2=79%, baixa certeza de evidência). Um estudo evidenciou melhores resultados da intubação bicanalicular com silicone em comparação a sondagem tardia no subgrupo das obstruções lacrimonasais congênitas complexas, (1 estudo; risco médio 0.56 [intervalo de confiança de 95% 0.34, 0.92] p=0,02, moderada certeza de evidência).

**Conclusões:**

Há evidências de baixa qualidade de que a sondagem precoce tem a mesma taxa de sucesso que a sondagem tardia. Evidências de moderada certeza sugerem que a sondagem tardia tem menor chance de sucesso do que a intubação bicanalicular com silicone em casos de obstruções lacrimonasais congênitas complexas.

## INTRODUCTION

Nasolacrimal duct obstruction (NLDO) is widespread in the pediatric population, occurring in up to 20% of newborns^(1)^. NLDO is usually congenital in origin and occurs due to a failure of canalization in the nasolacrimal duct^(2)^. The main symptoms of NLDO include epiphora, lash crusting, and reflux of mucopurulent discharge upon compression of the lacrimal sac^(3)^.

The natural history of NLDO is favorable, with resolution in most cases during the first year of life either spontaneously or after conservative treatment such as lacrimal sac massage^(4-6)^. When NLDO persists, lacrimal probing is the treatment of choice because it is relatively easy to perform^(7,8)^.

However, controversy exists with respect to the best time to probe. The decision to probe early (<12 months of age) versus late (>12 months) is usually based on the surgeon’s clinical judgment and experience. Some studies have reported a higher failure rate with late probing compared with early probing^(9-11)^. Studies have also reported a decrease in the success rate of lacrimal probing with an increase in the age of the child^(9-11)^. In complex cases, probing may be less effective than other more expensive therapies, such as lacrimal system intubation^(12)^.

A previous systematic review compared the success rates and complications of various types of NLDO treatment. However, this review included randomized controlled trials (RCTs) and non-randomized prospective studies, and did not use the Grading of Recommendations, Assessment, Development, and Evaluation (GRADE) classification system to evaluate the quality and certainty of the evidence^(13)^. A recently published Cochrane review, which included only two RCTs, concluded that the effect and cost of immediate versus deferred probing for NLDO remain uncertain for most outcomes^(14)^.

Therefore, we performed an updated systematic review of the literature to assess the effectiveness of probing compared with clinical observations or other treatments to treat congenital NLDO.

## METHODS

The methods used to perform this review were guided by the Cochrane Handbook for Intervention Reviews^(15)^. This systematic review was conducted by the Preferred Reporting Items for Systematic Reviews and Meta-Analyses (PRISMA) statement^(16)^.

### Eligibility criteria

RCTs and quasi-randomized studies that enrolled children up to 10 years old with congenital NLDO, irrespective of gender and etiology, were included. Interventions included office-based probing or hospital-based probing under general anesthesia. Studies included a control group that did not undergo probing (or in whom probing was deferred) or other interventions, including observation alone, antibiotic drops alone, antibiotic drops plus massage of the lacrimal sac (Crigler massage or emptying massage), canalicular intubation, dacryocystorhinostomy, endoscopic endonasal dacryocystorhinostomy, the association of two or more therapies, or no intervention.

The outcome measures included a primary outcome to report probing success, which was defined as the absence of clinical signs and symptoms of congenital NLDO. The secondary outcomes included the best time to perform lacrimal probing (early probing if patients were <12 months of age and late probing if patients were >12 months of age); the proportion of participants with anatomic and functional injuries due to probing (creation of a false passage and injury to the nasolacrimal duct, canaliculi, and puncta); quality of life; and cost (assessed narratively) of the intervention.

Animal studies, case series, cohort studies, case reports, and review articles were excluded from this review.

### Data source and searches

The following electronic databases were searched for relevant articles: the Cochrane Database of Clinical Trials (CENTRAL; 2019, issue 12); PubMed (1966 to December 2019); EMBASE (1980 to December 2019); the Latin American & Caribbean Health Sciences Literature (LILACS; 1982 to December 2019), and clinicaltrials.gov. Using Medical Subject Headings terms and free terms related to “congenital nasolacrimal duct obstruction,” “probing,” and “treatment,” the search strategy was replicated for CENTRAL, PubMed, EMBASE, LILACS, and clinicaltrials.gov (Appendix 1). There were no language or publication year restrictions. The search strategy was adapted for each database.

**Appendix 1:** Search strategy

[(nasolacrimal duct) or (nasolacrimal ducts) or (lacrimal duct Obstruction) or (lacrimal duct Obstructions) or (congenital nasolacrimal duct obstruction) or (congenital nasolacrimal ducts obstruction) and (probing) or(office probing) and (treatment) or (therapy)].

### Study selection and data extraction

The titles and abstracts were reviewed by two researchers to identify potentially relevant papers. The papers were obtained and independently read by two reviewers. If necessary, differences were resolved by consulting a third reviewer. Reasons for exclusion were identified. The data was also extracted independently by two reviewers based on a priori inclusion and exclusion criteria.

The following information was extracted: references (authors, setting, year of publication, study design, allocation generation, allocation concealment, blinding); patients (age, sex, number); intervention (type and time); follow-up period; and outcomes (measures of results and adverse effects).

### Risk of bias assessment

Two reviewers independently assessed the risk of bias in the RCTs using a modified version of the Cochrane Collaboration’s tool^(15)^, which includes nine domains: adequacy of sequence generation, allocation sequence concealment, blinding of participants and caregivers, blinding of data collectors, blinding for outcome assessment, blinding of data analysts, incomplete outcome data, selective outcome reporting, and the presence of other potential sources of bias not accounted for in the previously cited domains. When information was unavailable on the risk of bias or other aspects of the methods or results, the reviewers attempted to contact study authors for additional information.

### Certainty of evidence

The reviewers used the GRADE classification system for the certainty of evidence^(17)^. Each outcome was rated as either high, moderate, low, or very low. Detailed GRADE guidance was used to evaluate the overall risk of bias, imprecision, inconsistency, indirectness, and publication bias. The results were summarized in an evidence profile. If an outcome was subject to one or more of these factors, the reviewers downgraded the quality of the evidence from high to moderate, low, or very low depending on the number of reasons identified^(18,19)^.

### Data synthesis and statistical analysis

All outcomes were analyzed using dichotomous variables and pooled Mantel-Haenzel risk ratios (RRs) and associated 95% confidence intervals (CIs) using the random-effects models. The analyses were based on eligible patients who had reported outcomes in each study. Review Manager 5.3.5 software^(20)^ was used for all analyses.

If the results of the principal analysis reached statistical significance, the reviewers planned to conduct sensitivity analyses to test RCTs with a low risk of bias versus a high risk of bias, and withdrawal rates for each outcome were evaluated (i.e., <20% versus ≥20%).

Variability in the results was addressed using the I^^2^^ statistic and the p-value obtained from the chi-squared test for heterogeneity. Heterogeneity was considered when I^^2^^ >75%^(15)^. We performed a subgroup analysis according to the complexity of NLDO (simple vs. complex)^(12)^.

## RESULTS

### Study selection

**Figure 1:**
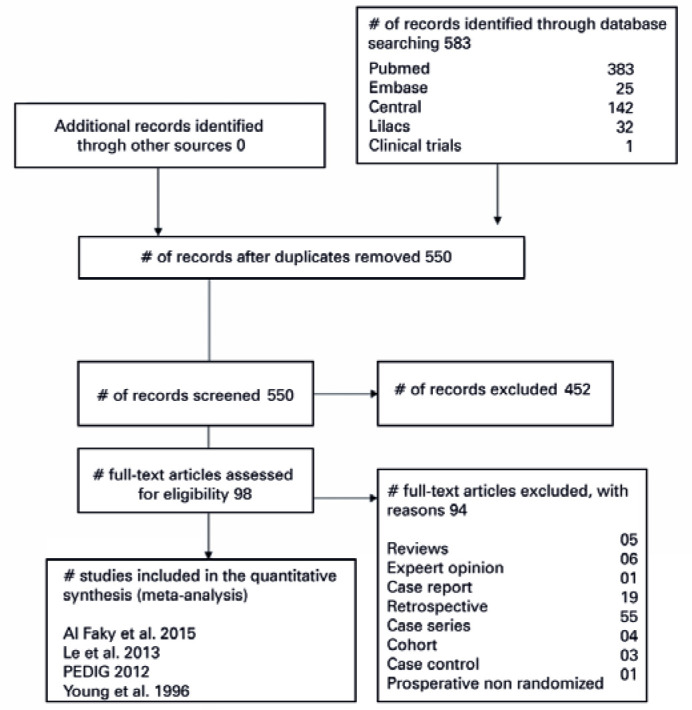
Review flowchart.

Figure 1 presents the process of identifying eligible studies. A total of 550 citations were identified after duplicates were removed. Based on screening of the title and abstract, 98 full texts were assessed, four of which were RCTs involving 423 participants (Al-Faky 2015, Lee 2013, Young 1996 e PEDIG 2012)^(12,21-23)^.

### Study characteristics

Table 1 describes the study characteristics such as design; country; the period of study and length of follow-up; number of participants; age; gender; inclusion and exclusion criteria; intervention; and outcomes. Two studies were conducted in the USA^(21,23)^, one in Saudi Arabia^(12)^, and one in the United Kingdom^(22)^. One study was a single-center study^(12)^ and the other three studies were multicenter studies^(21-23)^ This review includes 510 nasolacrimal ducts from 423 participants. The sample sizes of the RCTs ranged from 22^(22)^ to 181^(12)^ participants. Typical participants were infants aged from six months of life to 90 months. The follow-up period of the studies ranged from six months^(12)^ to two years^(22)^.

**Table 1:** Characteristics of included studies.

	Al-Faky 2015^(12)^	Lee 2013^(12)^	PED1G 2012^(23)^	Young 1996^(22)^
**Methods**	**Design: RCT^a^ Country:** Saudi Arabia (1 center) **Period:** Aug 2006 to Apr 2013 **Follow-up:** 6 months	**Design: RCT^a^ Country:** United States (22 centers) **Period:** Nov 2008 to Sep 2010 **Follow-up:** Up to 18 months old	**Design: RCT^a^** **Country:** USA (22 centers) **Period:** Nov 2008 to Sep 2010 **Follow-up:** Until age 18 months	**Design: RCT^a^** **Country:** United Kingdom (7 centers) **Follow-up:** Not reported
**Participants**	**Total:** 207 eyes (181 infants) **Age:** Probing group mean age: 27.4 ± 14.6 months; bicanalicular silastic intubation group mean age: 30.7 ± 15.5 months **Sex:** 49.7% girls; 50.3% boys	**Total: 1**14 eyes (57 infants) Age: from 6 to 10 months old (mean age 7.7 months) **Sex:** 42% girls and 58% boys	**Total:** 163 eyes (163 infants) Age: from 6 to 10 months old (mean age 7.7 months) **Sex:** 45.4% girls and 54.6% boys	**Total:** 26 eyes (22 infants) **Age:** Not reported, but infants were all “approaching or just after their first birthday” **Sex:** Not reported
**Inclusion criteria**	Children aged >1 year with epiphora and/or discharge before 6 months of age in absence of upper respiratory infection or ocular surface irritation. Enrolment for surgical treatment for the first time to treat NLDO was mandatory.	Children from 6 to 10 months old with bilateral NLDO (presence of epiphora, increased tear lake, and/or mucous discharge in both eyes); onset of symptoms before 6 months of age.	Onset of symptoms before 6 months of age; presence of at least one clinical sign of NLDO in the absence of an upper respiratory infection or ocular surface irritation; no prior nasolacrimal duct surgery.	Presenting within the time limits with no medical contraindication; NLDO with a history of epiphora and/ or discharge starting within 3 months of birth and an abnormal FDDT.
**Exclusion criteria**	Punctual disease; previous surgical intervention or acute dacryocystitis; eyelid malposition; Down syndrome; craniofacial anomaly; bony NLDO.	Patients with prior NLD surgery; Down syndrome; or craniofacial anomalies.	Children with Down syndrome or craniofacial anomalies.	History of previous lacrimal procedures.
**Intervention**	Probing after 1 year of age (88 patients) versus bicanalicular silastic intubation (93 patients).	Bilateral office-based NLD probing within two weeks of study entry (31 patients) versus 6 months of observation followed by probing for unresolved cases (26 patients).	Immediate office-based NLD probing (82 patients) versus 6 months of observation (81 patients) followed by for persistent symptoms.	Probing at 12 to 14 months of age (10 NLD) versus no treatment until 24 months (16 NLDs).
**Outcomes**	Resolution of all preoperative manifestations; normal FDDT; and positive Jones primary dye test.	Absence of clinical signs and symptoms of NLDO.	Absence of clinical signs and symptoms of NLDO.	Complete or near complete remission of symptoms and signs and a normal FDDT.

RCT= randomized clinical trials; NLDO= nasolacrimal duct obstruction; NLD= nasolacrimal duct; FDDT= fluorescein disappearance dye test.

### Risk of bias in the included studies

**Figure 2:**
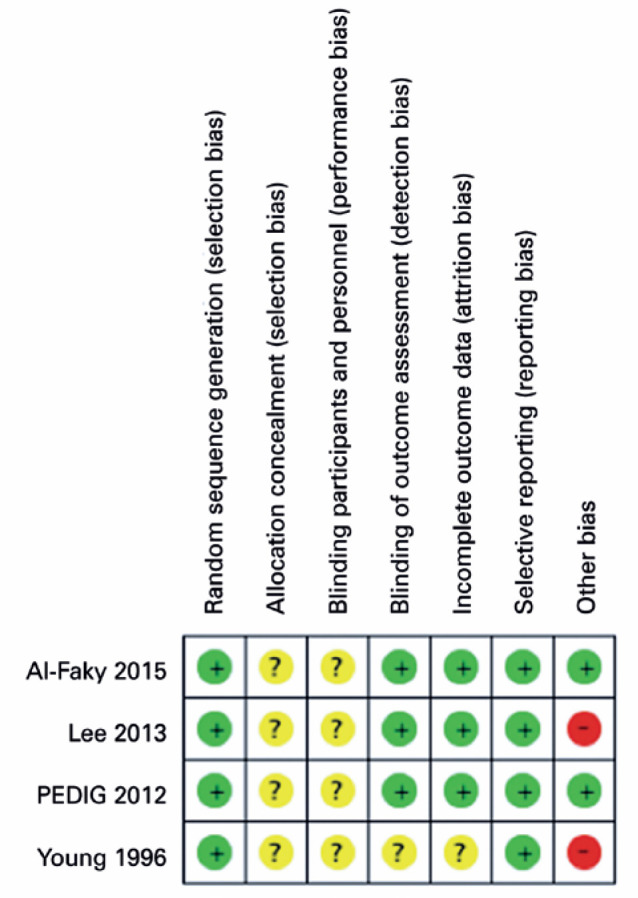
Risk of bias summary. Review authors’ judgments about each risk of bias item for each study included in the meta-analysis.

The risk of bias in the four individual studies included in the review and judgments is presented in figure 2. The major issue in relation to the risk of bias was due to lack of information about allocation concealment and blinding of participants and personnel^(12,21-23)^.

### Outcomes

**Figure 3:**
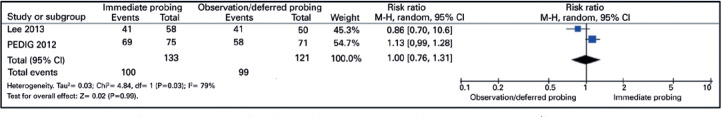
Meta-analysis. Resolution rate of congenital nasolacrimal duct obstruction: early probing vs. observation/ late probing according to the number of nasolacrimal ducts. CI, confidence interval; p< 0.05 was considered statistically significant.

**Figure 4:**
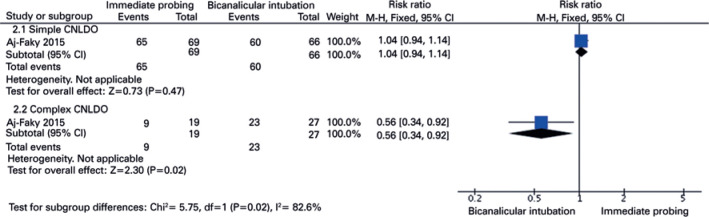
Resolution rate of congenital nasolacrimal duct obstruction. Late probing vs. bicanalicular silastic intubation according to the complexity of nasolacrimal duct obstruction.

Results from two RCTs^(21,23)^ suggested no statistical difference between early probing compared with observation/late probing in the congenital NLDO resolution rate (RR 1.00 [CI 95% 0.76-1.33]; p=0.99; I^^2^^=79%) (Figure 3). Concerning the resolution rate of congenital NLDO between late probing and bicanalicular silastic intubation, according to the complexity of obstruction (Figure 4), results from one RCT in the subgroup of interest suggested a statistical difference, which favored the bicanalicular silastic intubation in complex congenital NLDO.

### Intervention effects

Tables 2 and 3 contain the results of the GRADE classi fication of the certainty of evidence.

**Table 2:** Summary of findings for the comparison of early probing vs. observation/late probing for congenital nasolacrimal duct obstruction.

Early probing compared with observation/late probing for congenital nasolacrimal duct obstruction Patient or population: children with congenital nasolacrimal duct obstruction (CNLDO) Context: community-based population in the USA Intervention: early probing Comparison: observation/late probing if needed						
Outcomes	**Anticipated absolute effects (95% CI)**		**Relative effect (95% CI)**	**N^o^ of participants (studies)**	**Certainty of the evidence (GRADE)**	**Comments**
	**Risk with observation/ late probing**	Risk with early probing				
**Resolution of CNLDO according to NLDs** (follow-up: 9 to 12 months)	**818 per 1,000**	**818 per 1,000 (622 to 1000)**	**RR 1.00** (0.76 to 1.31)	254 (2 RCTs)	⊕⊕⊝⊝ **Low** ^+,++^	Risk estimates based on PEDIG, 2012^(23)^ study (largest trial).

* The basis for the assumed risk is the mean control group risk. The corresponding risk (and its 95% confidence interval) is based on the assumed risk in the comparison group and the relative effect of the intervention (and its 95% CI).

CI= confidence interval; RR= risk ratio; CNLDO= congenital nasolacrimal duct obstruction; OR= Odds ratio; NLD= nasolacrimal duct; RCT= randomized clinical trial.

**GRADE working group grades of evidence.**

**High certainty:** Further research is very unlikely to change our confidence in the estimate of the effect.

**Moderate certainty:** Further research is likely to have an important impact on our confidence in the estimate of the effect and may change the estimate.

**Low certainty:** Further research is very likely to have an important impact on our confidence in the estimate of the effect and is likely to change the estimate.

**Very low certainty:** We are very uncertain about the estimate.

+ Downgrade for imprecision because CI 95% for absolute effects inclsuded clinically important benefit and no benefit. In addition, the sample size was small and did not reach CI 95%.

++ Downgrade of inconsistency because I^^2^^ = 79%.

**Table 3:** Summary of findings for the comparison late probing vs. bicanalicular silastic intubation for congenital nasolacrimal duct obstruction

Late probing compared with bicanalicular silastic intubation for CNLDO. Patient or population: children with congenital nasolacrimal duct obstruction (CNLDO) Context: community-based population in the Saudi Arabia Intervention: late probing Comparison: bicanalicular silastic intubation						
**Outcome**	**Anticipated absolute effects (95% CI)**		**Relative effect (95% CI)**	**N** ^o^ **of participants (studies)**	**Certainty of the evidence (GRADE)**	**Comments**
	**Risk with bicanalicular silastic intubation**	**Risk with late probing**				
**Resolution of CNLDO according to complexity 1) Simple CNLDO 2) Complex CNLDO** (follow-up: 6 months)	**909 per 1,000**	**945 per 1000** (855 to 1000)	**RR 1.0** (0.94 to 1.14)	135 (1 RCT)	⊕⊕⊕⊝ **Moderate**^+^	Risk estimates based on Al-Faky et al. 2015.^(12)^
	**852 per 1,000**	**477 per 1000** (290 to 784)	**RR 0.56** (0.34 to 0.92)	46 (1 RCT)		

* The basis for the **assumed risk** is the mean control group risk. The **corresponding risk** (and its 95% CI) is based on the assumed risk in the comparison group and the **relative effect** of the intervention (and its 95% CI).

CI= confidence interval; RR= risk ratio; CNLDO= congenital nasolacrimal duct obstruction; OR= Odds ratio; NLD= nasolacrimal duct; RCT= randomized clinical trial

**GRADE working group grades of evidence**

**High certainty:** Further research is very unlikely to change our confidence in the estimate of the effect.

**Moderate certainty:** Further research is likely to have an important impact on our confidence in the estimate of the effect and may change the estimate.

**Low certainty:** Further research is very likely to have an important impact on our confidence in the estimate of the effect and is likely to change the estimate. **Very low certainty:** We are very uncertain about the estimate.

^+^ Downgrade for imprecision because CI 95% for absolute effects included clinically important benefit and no benefit. In addition, the sample size was small and did not reach CI 95%.

## DISCUSSION

### Main findings

The present review was performed to address the divergence of opinion on the treatment of congenital NLDO, especially the need for early probing in children. The study indicates that the primary outcomes (treatment success; resolution rate) did not differ between early and late probing when performed before 16 months of age. Therefore, the success rate of probing does not decrease when the procedure is performed up to 16 months of age.

Many authors advocate clinical observation as the best option for congenital NLDO since 70% to 90% of obstructions may resolve spontaneously with conservative treatment using lacrimal sac massage in the first year of life^(23-27)^. Probing should be reserved for non-regression cases because it is a simple, safe, and effective procedure. Other studies suggest early probing to reduce symptoms and mitigate the risk of major complications of congenital NLDO, such as chronic inflammation, fibrosis, and infection, which worsen disease prognosis^(28-30)^.

The absence of differences between interventions (early probing vs. clinical observation/late probing) demonstrated in this meta-analysis is important to guide the surgeon’s decision about the best treatment logistics, improving clinical care for patients with congenital NLDO. Also, it allows the consideration of other factors related to lacrimal probing, such as the risks involved in general anesthesia (necessary for older children) and the cost of the procedure.

Regarding the cost-effectiveness of late probing to treat congenital NLDO, the PEDIG study^(23)^ reported a 20% increase in the final cost, including the expenses of an initial office consultation and all medications prescribed and surgeries received. According to the authors of this study, although unilateral congenital NLDO often resolves without surgery, immediate office probing is an effective and potentially cost-saving treatment option^(23)^.

Interesting evidence for clinical practice, which should be confirmed by new studies, suggests the superiority of bicanalicular silastic intubation over late probing for complex obstructions^(12)^. Intubation is a complex and expensive procedure, which mostly requires general anesthesia and insertion of a stent device. Conversely, probing is simple, quick, and inexpensive. However, with complex congenital NLDO, there is greater difficulty in recanalization of the lacrimal pathway, justifying the cost of intubation and anesthetic risk.

### Relation to prior work

Two systematic reviews^(13,14)^, which are relevant to our study objectives, have been published in recent years. Lin et al.^(13)^ included seven studies, four RCTs, and three prospective non-randomized studies. They compared the success rates and complications of various types of congenital NLDO treatment besides probing, and concluded that success rates did not differ between immediate and deferred probing; between balloon dilation and intubation; and between monocanalicular and bicanalicular intubation. However, a review by Lin et al.^(13)^ presented limitations related to the inclusion of non-randomized prospective studies, which lower the quality and relevance of the results. It is well known that non-randomized studies are prone to confusion because interventions are often prescribed to patients based on the perceived risk of the outcomes rather than being randomly assigned, as in RCTs^(31,32)^. Also, Lin et al.^(13)^ did not use the GRADE system to assess the quality and strength of evidence.

Another review published by the Cochrane Collaboration^(14)^ included two RCTs but used the GRADE system to qualitatively evaluate one study and did not perform a meta-analysis. It concluded that there is no clear difference between immediate probing and observation alone for the resolution of congenital NLDO, and that immediate probing may be more beneficial than late probing for unilateral obstruction.

Thus, the results of this review overlap with those of the previous two reviews; however, our findings provide a higher level of evidence, as they are based on a meta-analysis of RCTs.

### Strengths and limitations

The present review has numerous strengths, including an extensive and sensitive search of the literature with no restrictions on language or publication status. The analysis of risk factors for bias in the included studies, which followed strict Cochrane Collaboration assessment standards, indicated a low risk of bias and good methodological quality. The only exception was in the study by Young et al.^(22)^, which presented an uncertain risk of bias.

In addition to the methodological evaluation, the present review utilized the GRADE system, which has been used by several international institutions to classify the strength of the recommendation of health evidence. Among these institutions are the World Health Organization, the National Institute for Health and Care Excellence (NICE), the Centers for Disease Control and Prevention (CDC), and the Cochrane Collaboration.

A limitation of this review was the small number of studies included and the high heterogeneity observed in the meta-analysis (79%). The small sample size, surgeons’ different levels of experience, and individual patient characteristics may have contributed to heterogeneity. However, as studies by Lee et al.^(21)^ and PEDIG^(23)^ were based on the same protocol and were therefore methodologically similar, heterogeneity can be considered inexplicable. These findings reinforce the need for additional homogeneous studies.

The certainty of evidence of the primary outcome, resolution rate of congenital NLDO, was low; therefore, future research will likely have a significant impact on confidence when estimating the effect of the intervention. The outcomes of the research are likely to alter the estimate^(18)^. This rate was due to serious imprecision (small sample size and wide CIs) and inconsistency (unexplained heterogeneity). In the secondary outcomes (resolution rate of congenital NLDO in complex obstructions), the certainty of evidence was classified as moderate due to imprecision (restricted sample size and wide CIs).

The evaluation of GRADE in this review revealed that the strength of recommendation of the evidence on the effectiveness of probing in congenital NLDO must improve, and new studies with greater standardization and larger sample sizes are required to draw definitive conclusions.

In the treatment of congenital NLDO, early probing performed from six months of age until ten months of age results in an equivalent chance of therapeutic success when compared with late probing performed between 12 months and 16 months of age (low certainty of evidence). There is evidence that late probing has a lower chance of success compared with bicanalicular silastic intubation for complex congenital NLDO (moderate certainty of evidence).

### Implications for clinical practice

Due to the evidence found in this review, specialists can wait for a spontaneous resolution of congenital NLDO or proceed to probing without risk of worsening the prognosis due to therapeutic choice. This decision will depend on the experience of each ophthalmologist and should be discussed with parents/guardians to ensure optimal treatment in each case. Additionally, it is important to consider the risks inherent in the procedures and the costs involved.

### Implications for the research

Further RCTs with methodological quality, standardized endpoints, and larger sample sizes are needed to confirm the effectiveness of probing in congenital NLDO and to reinforce the strength of the evidence in the literature to provide robust outcome estimates. Further research is needed to provide a better understanding of the role of probing in the treatment of congenital NLDO.
